# External rotation elastic bands at the lower limb decrease rearfoot eversion during walking: a preliminary proof of concept

**DOI:** 10.1590/bjpt-rbf.2014.0194

**Published:** 2016-11-16

**Authors:** Thales R. Souza, Vanessa L. Araújo, Paula L. Silva, Viviane O. C. Carvalhais, Renan A. Resende, Sérgio T. Fonseca

**Affiliations:** 1 Departamento de Fisioterapia, Universidade Federal de Minas Gerais (UFMG), Belo Horizonte, MG, Brazil; 2 Department of Psychology, University of Cincinnati, Cincinnati, OH, United States of America

**Keywords:** walking, coupling, proximal influence, elastic band, rearfoot, movement

## Abstract

**Background:**

Reducing rearfoot eversion is a commonly desired effect in clinical practice to prevent or treat musculoskeletal dysfunction. Interventions that pull the lower limb into external rotation may reduce rearfoot eversion.

**Objective:**

This study investigated whether the use of external rotation elastic bands, of different levels of stiffness, will decrease rearfoot eversion during walking. We hypothesized that the use of elastic bands would decrease rearfoot eversion and that the greater the band stiffness, the greater the eversion reduction.

**Method:**

Seventeen healthy participants underwent three-dimensional kinematic analysis of the rearfoot and shank. The participants walked on a treadmill with and without high- and low-stiffness bands. Frontal-plane kinematics of the rearfoot-shank joint complex was obtained during the stance phase of walking. Repeated-measures ANOVAs were used to compare discrete variables that described rearfoot eversion-inversion: mean eversion-inversion; eversion peak; and eversion-inversion range of motion.

**Results:**

The low-stiffness and high-stiffness bands significantly decreased eversion and increased mean eversion-inversion (p≤0.037) and eversion peak (p≤0.006) compared with the control condition. Both bands also decreased eversion-inversion range of motion (p≤0.047) compared with control by reducing eversion. The high-stiffness band condition was not significantly different from the low-stiffness band condition for any variables (*p*≥0.479).

**Conclusion:**

The results indicated that the external rotation bands decreased rearfoot eversion during walking. This constitutes preliminary experimental evidence suggesting that increasing external rotation moments at the lower limb may reduce rearfoot eversion, which needs further testing.

## Bullet Points

- Acute use of external rotation bands reduces rearfoot eversion during walking.
- The aim of the interventions was to pull the limb into external rotation.
- As this is preliminary evidence, implications should be considered with caution.

## Introduction

Proximal lower limb mechanics in the transverse plane and foot pronation-supination (commonly measured as rearfoot eversion-inversion) seem to be coupled during the stance phase of walking^1^ ^,^ ^2^. This coupling has been considered in multifactorial approaches to musculoskeletal painful conditions^3^ ^,^ ^4^, such as foot-ankle and knee disorders possibly related to abnormal pronation and supination^5^ ^-^ ^9^. Although influenced by transverse-plane motion at the knee^10^, coupling of rearfoot eversion-inversion with lower limb internal-external rotation during walking^1^ ^,^ ^2^ may enable forces at the foot to change both rearfoot and lower limb kinematics^10^ ^,^ ^11^ in a distal to proximal direction. Accordingly, a proximal to distal effect during walking is expected in which forces at joints proximal to the ankle, acting on the transverse plane, influence shank axial rotation^12^ ^,^ ^13^ and may affect rearfoot eversion-inversion^14^.

According to the coupling described, mechanisms that pull the lower limb into external rotation, such as the action of external rotator muscles and other tissues at the thigh and shank, may decrease rearfoot eversion during walking stance^3^ ^,^ ^14^. Thus, interventions aimed at increasing external rotation moments at the lower limb can be proposed to reduce rearfoot eversion^15^. External rotation elastic bands wrapped around the pelvis and lower limb^16^ can be used to test for this possible effect on rearfoot kinematics.

The present study investigated this effect by using elastic bands designed to pull thigh and shank into external rotation, as a proof of concept. We hypothesized that the use of the bands would decrease rearfoot eversion at the foot-ankle complex. We also hypothesized that a stiffer elastic band would cause greater decreases in eversion than a less stiff band.

## Method

### Participants

Seventeen healthy participants (11 females, 6 males) participated in the study (26.5±2.59 years, 62.62±7.84 kg, 168.0±8.0 cm). They were symptom-free, did not have any pathologic condition in the lower limbs or lumbo-pelvic complex during the previous six months, and had never undergone orthopedic surgery. The participants had a maximum body mass index of 25 Kg/m^2^ and had never used any type of foot orthosis. They had a maximum foot-shank angle of 24°^17^, passive range of motion of hip internal rotation between 23° and 71° and passive range of motion of hip external rotation between 25° and 56°^18^. The Ethics Committee of Universidade Federal de Minas Gerais (UFMG), Belo Horizonte, MG, Brazil approved this study (protocol number: CAAE – 0427.0.203.000-11) and all participants signed informed consent forms.

### Elastic bands

**Figure 1:**
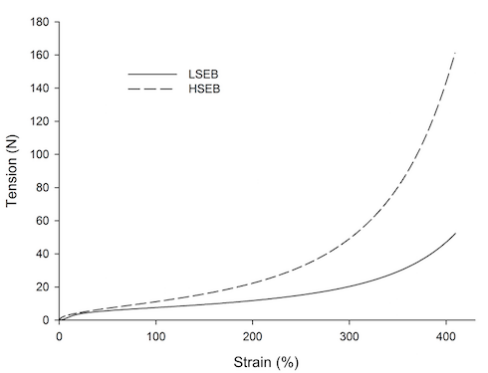
Tension (Newtons) versus strain (deformation in %) relationships describing each elastic band. LSEB: Low-stiffness elastic band; HSEB: High-stiffness elastic band.

**Figure 2:**
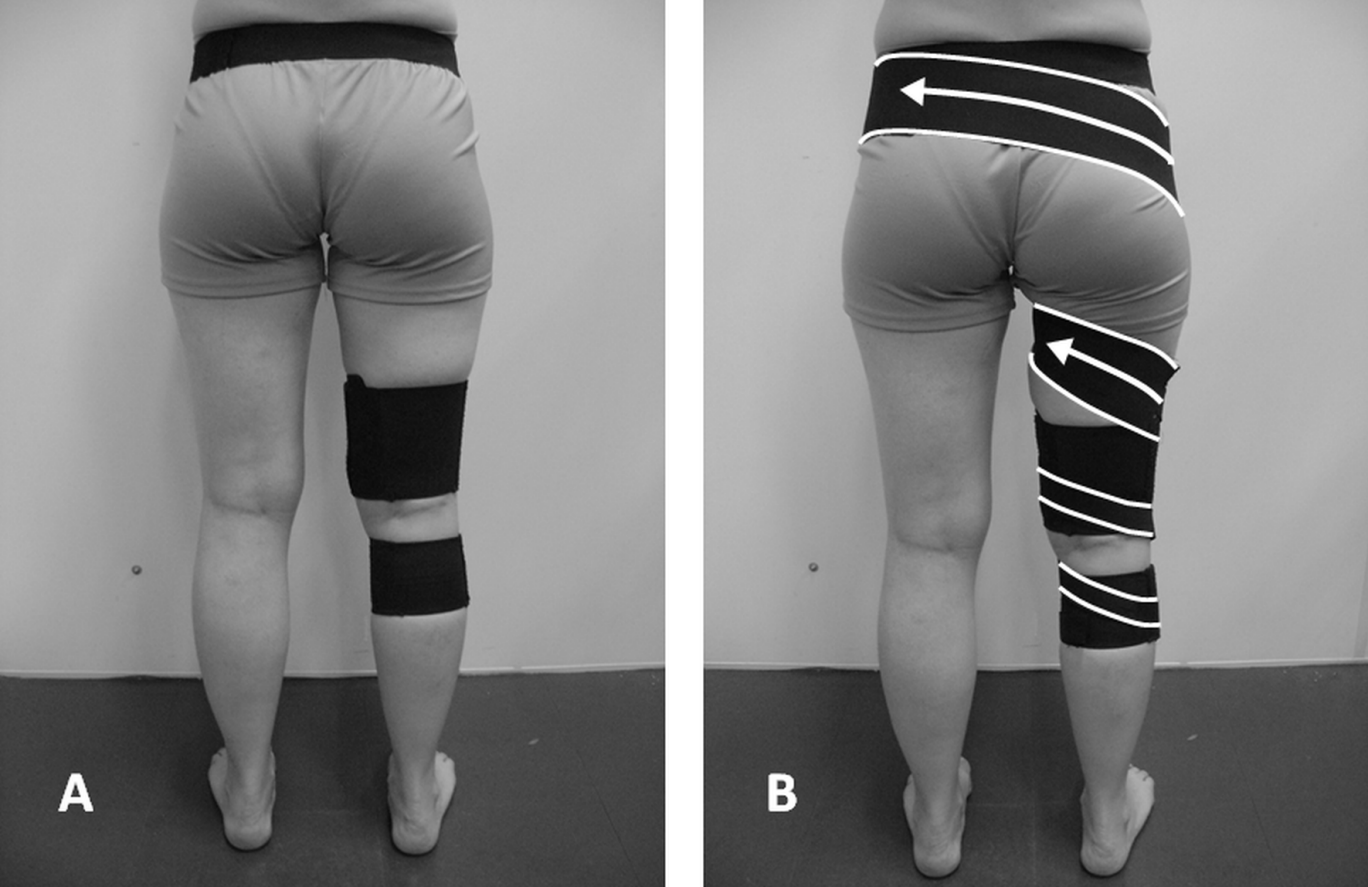
(A) Elastic belts. (B) Elastic band attached to the elastic belts. Limits of the elastic band are highlighted in white. Arrows indicate the tension direction in the elastic portion of the band.

Two elastic bands were used to pull the lower limb into external rotation: one low-stiffness elastic band (LSEB) and one high-stiffness elastic band (HSEB). The stress-strain relationships of the bands are presented in Figure 1. Each band was attached to three elastic belts, which were firmly fastened with Velcro^®^ to 1) the iliac bones of the pelvis, 2) the distal third of the thigh, and 3) the proximal third of the shank (Figure 2A). The shank belt was used to improve the attachment of the elastic band to the lower limb, since the thigh belt is prone to large displacements due to the high volume of soft tissues. To produce external rotation, one of the elastic bands was wrapped around the lower limb in a spiral fashion, from the lateral aspect of the pelvic belt (contralateral to the lower limb studied) to the lateral aspect of the thigh and shank belts (Figure 2B). The proximal end of the band was attached to the pelvic belt and the distal end was attached to the thigh and shank belts with Velcro^®^. The middle portion of the elastic band passed through the posterior aspect of the pelvis, across the hip joint, and around the thigh. This portion passed over the greater trochanter to avoid affecting frontal-plane moments at the hip. Each band was wrapped around the limb with the elastic portion stretched, with a deformation of 20 cm (35.7%). This amount of strain guaranteed that the elastic bands would not slacken during walking. The bands were wrapped around the lower limb while the participant was in bipedal stance. All participants reported that the bands, when attached and stretched, pulled the limb into external rotation.

### Foot kinematics

#### Procedure

**Figure 3:**
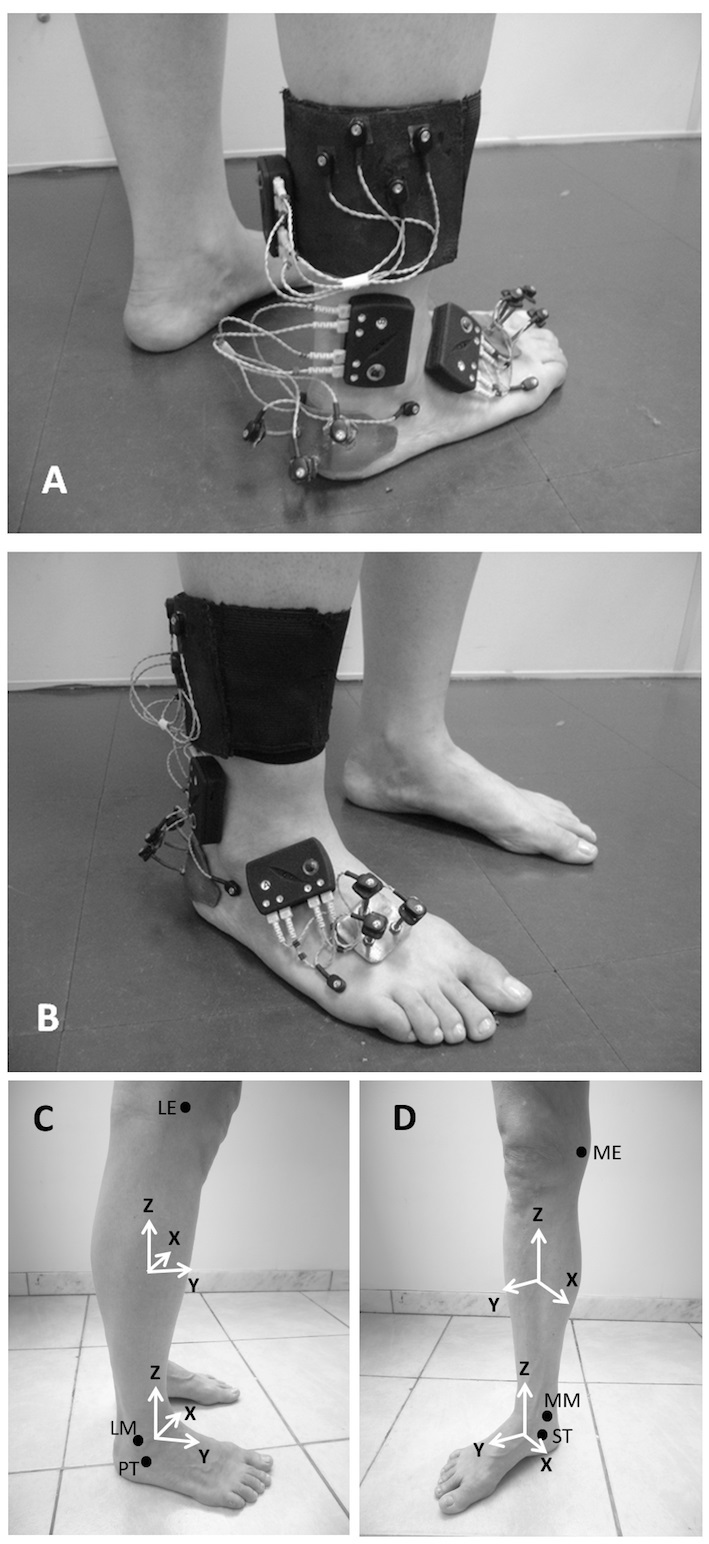
Clustered active tracking markers (A and B), coordinate systems of the segments, and anatomical references (black circles) (C and D). Technical markers on the peroneal tubercle and fifth metatarsal head are shown, as well as the drive boxes and cables of the markers. The cluster on the forefoot was not used in the present study. ME: medial epicondyle; LE: lateral epicondyle; MM: medial malleolus; LM: lateral malleolus; PT: peroneal tubercle; ST: sustentaculum tali.

A kinematic analysis of the rearfoot-ankle complex^19^ was implemented with the Codamotion three-dimensional analysis system (Charnwood Dynamics Ltd., Rothley, England), with three scanner units and active markers. The sampling rate was set at 100 Hz. Clusters of tracking markers were used to determine the displacement of each segment (Figure 3A and B). The shank cluster consisted of an elastic belt with a rigid plate to which four tracking markers were attached. This cluster was attached to the distal third of the shank^20^. The rearfoot cluster consisted of flexible metallic bases, each with three rigid rods to which tracking markers were attached. The rearfoot cluster was attached to the posterior aspect of the calcaneus, below the insertion of the calcaneus tendon^19^. Two technical markers were attached to the lateral aspect of the foot – one on the peroneal tubercle of the calcaneus and the other on the fifth metatarsal head – for posterior definition of the stance phase of walking^21^.

A static trial was conducted with the participant in relaxed bipedal standing without the elastic bands. Following this, the participants were required to walk on an electric treadmill ProAction G635 Explorer (BH Fitness, Vitoria-Gasteiz, Spain), at their preferred speed, in three conditions: (1) control condition - without elastic bands; (2) LSEB condition - with the low-stiffness elastic band; and (3) the HSEB condition - with the high-stiffness elastic band. The sequence of conditions was randomized. A trial with at least 20 steps of the right limb was registered in each condition. Before the walking trials, the participants were allowed to walk freely in each condition for approximately five minutes to familiarize themselves with the use of the elastic bands and segment belts. The experimental conditions and measures were always conducted on the right limb in order to standardize the laboratory setup.

#### Data reduction

Data processing was carried out using Visual 3D software (C-Motion Inc., Rockville, MA, USA). A global coordinate system (X,Y,Z) was created using the medial-lateral and anterior-posterior orientations of the treadmill as the X and Y axes, respectively. The global longitudinal axis (Z axis) was orthogonal to the X and Y axes.

A six-degrees-of-freedom (6DOF) kinematic model was used, in which the segments were assumed to be free rigid bodies^22^. According to this model, anatomical references were used to determine a local coordinate system for each segment: shank and rearfoot (Figure 3C and D)^19^. During the static trial, a pointer was used to define digitally the location of the anatomical references of the participant within the global coordinate system. Two proximal references and two distal references, lateral and medial, were used for each segment. The proximal references of the shank were the lateral and medial epicondyles of the femur, and the distal references were the lateral and medial malleoli. The proximal references of the rearfoot were the malleoli, and the distal references were the peroneal tubercle and sustentaculum tali. We used the standard method of the Visual 3D software to create the coordinate systems of each segment. The longitudinal axis (Z axis) was defined as the line connecting the midpoint between the proximal references to the midpoint between the distal references. The medial-lateral axis (X axis) was defined as the line minimally distant from a lateral-medial line connecting the proximal references and a lateral-medial line connecting the distal references. It was calculated using the least-squares fit method. The Y axis (posterior-anterior) of each segment was orthogonal to the axes previously created. The clusters of tracking markers were digitally associated with the corresponding coordinate systems (segments) so that the position variation of these markers determined the position variation of the segments.

The stance phase of walking was defined as the period between initial contact and toe-off. These events were determined using the linear anterior-posterior motion (in the global Y axis) of the technical markers^21^. We plotted the curves of the markers’ linear motion to facilitate event identification. Initial contacts were defined as the instants in which forward motion of the rearfoot marker stopped, which corresponded to the peaks of the corresponding curve. Toe-offs were defined as the instants in which backward motion of the forefoot marker stopped, which corresponded to the valleys of the corresponding curve. Two examiners determined these events. To test the reliability of this method, we carried out a pilot study with ten participants and two evaluation sessions separated by a one-week interval. We observed intraclass correlation coefficients greater than 0.99 for the intra- and inter-examiner reliabilities^23^.

The motion of the rearfoot relative to shank (rearfoot-shank) was calculated, in the frontal plane (around the segments Y axes), for the stance phase of walking. The Cardan/Euler sequence used was: sagittal, frontal, and transverse^24^. The data were low-pass filtered with a zero-lag Butterworth filter and a cut-off frequency of 6 Hz^25^. The neutral positions (0°) for the angles obtained were defined as the positions recorded during the initial static trial in a relaxed standing position. Curves of angular position relative to stance time were plotted for 20 stance phases of each participant, from which discrete variables were calculated and used for statistical analyses. Mean curves corresponding to angular positions relative to percentage of stance were also obtained for each participant in each study condition for descriptive purposes and to test between-trials reliability of the time series obtained^26^. Reliability was tested through coefficients of multiple correlation (CMC), for two randomly selected stance phases in the control condition^26^. A mean CMC of 0.95 (SD 0.03) was found.

#### Variables definition

Discrete variables were obtained to represent specific features of the eversion-inversion curve of the rearfoot relative to the shank: (a) Mean eversion-inversion, to represent a position trend during the whole stance phase; (b) Eversion peak, which consists of the maximum value of rearfoot eversion; and (c) Eversion-inversion range of motion, which consists of the total range of motion. These discrete variables were calculated from each curve (i.e., 20 stance phases) and then averaged for each participant at each study condition. Between-trials reliability of these variables was calculated using Intraclass Correlation Coefficients (ICC) and standard error of measurements (SEM)^23^. For reliability testing, two randomly selected stance phases of each participant in the control condition were used. The ICC values obtained were 0.93, 0.95, and 0.92 for mean eversion-inversion, eversion peak, and eversion-inversion range of motion, respectively. The SEM values obtained were 0.97°, 0.77°, and 1.29° for mean eversion-inversion, eversion peak, and eversion-inversion range of motion, respectively.

### Statistical analysis

Repeated-measures analyses of variance (ANOVAs) with one factor (study conditions) and three levels (control, LSEB, and HSEB) were used to investigate differences among the study conditions. One ANOVA was carried out for each outcome variable. When the main effect was significant, pre-planned contrasts (control vs LSEB; control vs HSEB; LSEB vs HSEB) were used for pairwise comparisons. The alpha level was set at 0.05 for all analyses.

## Results

The ANOVA main effect was significant for mean eversion-inversion (p=0.003; F=7.83; $\eta^{2}$=0.3). Contrasts revealed that mean eversion-inversion was significantly greater (i.e., presenting less everted positions) in the LSEB (p<0.001; F=19.64; $\eta^{2}$=0.55) and HSEB (p=0.037; F=5.17; $\eta^{2}$=0.24) conditions compared with control. Mean eversion-inversion in the LSEB condition was not significantly different compared with the HSEB condition (p=0.479; F=0.52; $\eta^{2}$=0.03).

For eversion peak, the ANOVA main effect was significant (p<0.001; F=11.31; $\eta^{2}$=0.41). Contrasts revealed that the values of eversion peak were significantly greater (i.e., less everted peak positions) in the LSEB (p=0.001; F=18.2; $\eta^{2}$=0.53) and HSEB (p=0.006; F=9.8; $\eta^{2}$=0.38) conditions compared with control. Eversion peak in the LSEB condition was not significantly different compared with the HSEB condition (p=0.649; F=0.21; $\eta^{2}$=0.01).

For eversion-inversion range of motion, the ANOVA main effect was significant (p<0.012; F=5.16; $\eta^{2}$=0.24). Contrasts revealed that eversion-inversion range of motion was significantly smaller in the LSEB (p=0.022; F=6.47; $\eta^{2}$=0.28) and HSEB (p=0.047; F=4.61; $\eta^{2}$=0.22) conditions compared with control. Eversion peak in the LSEB was not significantly different compared with the HSEB condition (p=0.856; F=0.03; $\eta^{2}$=0.002).

**Figure 4:**
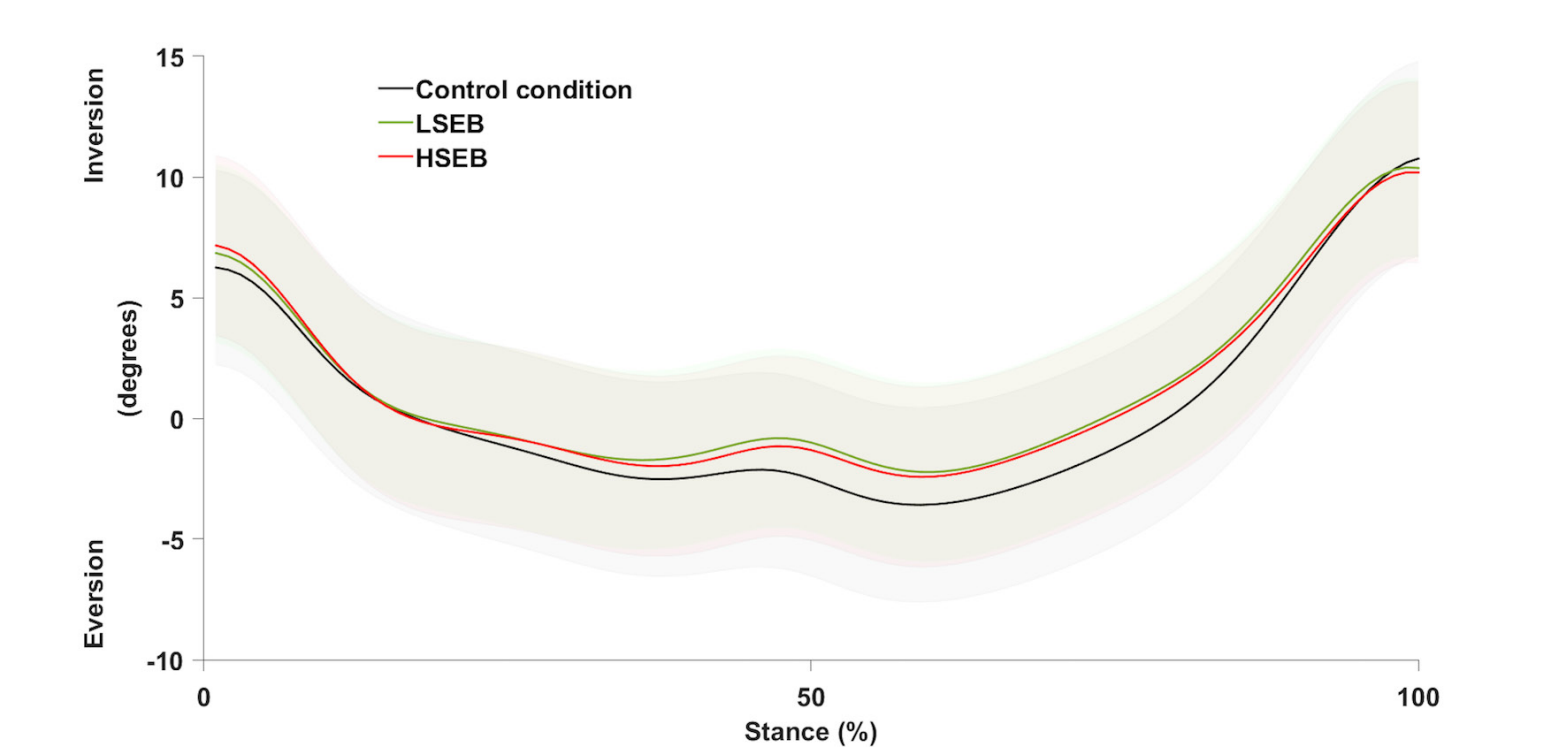
Average curves and standard deviations (shaded areas) of rearfoot-shank eversion-inversion angles during the stance phase of walking, in each experimental condition. LSEB: Low-stiffness elastic band condition; HSEB: High-stiffness elastic band condition.

The mean values, standard deviations, and value ranges of these outcome variables, in each experimental condition, are presented in Table 1. The curves showing the mean values of rearfoot eversion-inversion for all participants in each experimental condition are presented in Figure 4.

**Table 1:** Mean values, standard deviations, and ranges of the outcome variables in the experimental conditions.

	**Control** Mean (SD) [Range]	**LSEB** Mean (SD) [Range]	**HSEB** Mean (SD) [Range]
**Mean eversion-inversion**	0.49º (3.69) [-9.06º to 5.91º]	1.32º (3.76)* [-8.25º to 7.02º]	1.16º (3.96)* [-8.02º to 7.19º]
**Eversion peak**	-4.16º (3.45) [-12.45º to 0.40º]	-3.02º (3.37)* [-11.32 to 1.87º]	-3.10º (3.73)* [-11.49º to 1.65º]
**Eversion-inversion range of motion**	15.60º (4.59) [7.12º to 26.14º]	14.22º (3.29)* [6.60º to 18.70º]	14.26º (3.26)* [5.92º to 17.95º]

LSEB: Low-Stiffness Elastic Band condition; HSEB: High-Stiffness Elastic Band condition; SD: standard deviation.

* Significantly different from control condition (p≤0.05).

## Discussion

The use of external rotation elastic bands at the lower limb significantly changed mean eversion-inversion, eversion peak, and eversion-inversion range of motion of the rearfoot relative to the shank, which indicates a reduction in foot pronation and supports the first hypothesis of the study^3^ ^,^ ^27^ ^,^ ^28^. These results constitute preliminary experimental evidence that suggest an isolated contribution of proximal transverse-plane mechanics to rearfoot frontal-plane kinematics during walking. It agrees with recent findings of a correlation between hip passive resistance to internal rotation and rearfoot frontal-plane kinematics^14^. Nevertheless, the absence of measures of hip and knee motion or moments of force precludes making a strong conclusion on the proximal mechanical effects as causing the kinematic changes at the rearfoot, which motivates further testing.

Reductions in rearfoot eversion range of motion tended to occur mainly within the midstance and terminal stance phase of gait (i.e., approximately from 25% and 85% of the stance phase) (Figure 4). Thus, the effects of the bands seem to be evident in these sub-phases and manifested as decreases in maximum everted positions as well as increases in subsequent inversion motion. The decreases in range of motion also indicated decreases in eversion since the initial and final positions in the stance phase were very similar among conditions (Figure 4). The present findings point to potential effects that interventions aimed at increasing proximal external rotation moments at the lower limb have on rearfoot kinematics. The magnitudes of the kinematic changes observed in the rearfoot are similar to those related to interventions that proved to be beneficial in clinical situations, such as foot orthoses^29^ ^,^ ^30^. However, it should be recognized that any potential clinical effects of this type of intervention are still highly speculative and warrant future investigation.

The second hypothesis of the study was that eversion reductions would be greater with the HSEB than with the LSEB. However, the effects of the bands were not significantly different. Because the bands also act during the swing phase, the lower extremity and foot could be more externally rotated since the beginning of stance, particularly in the HSEB condition. A more externally rotated foot at initial contact leads to increases in eversion^31^. Thus, a possible explanation for the lack of differences between the bands is that, in the HSEB condition, the eversion moments of force produced by the ground reaction force on the externally rotated foot were great enough to reduce the range of motion of foot supination caused by the use of the band. To verify this possibility, we carried out a post-hoc repeated-measures ANOVA to compare the transverse-plane position of the foot at initial contact (represented by the rearfoot), relative to the ground, among the experimental conditions (main-effect p<0.001, F=16.37). LSEB and HSEB increased foot external rotation, in comparison with control (p≤0.013, F≥7.79). However, there was not a significant difference between band conditions (p=0.109, F=2.88). It should be noted that, although the effect size of this last comparison ($\eta^{2}$=0.42) can be considered large^32^, the statistical power was only 0.63. This indicates that the sample size of the study was not sufficient for this specific post-hoc comparison^23^. Therefore, we cannot draw a final conclusion at this point about the possible influence of foot external rotation on the similar results observed with the HSEB and the LSEB. Importantly, it should be stressed that due to the absence of measures of moments at the lower limb joints, we cannot assume that the bands actually produced different moment magnitudes. Thus, the similar effects of the bands on rearfoot kinematics may also be due to similar external rotation moments generated by the bands.

Previous studies investigated the influences of proximal mechanics on rearfoot eversion-inversion^15^ ^,^ ^33^, but not of isolated transverse-plane mechanics. Snyder et al.^15^ found that strengthening hip abductor and external rotator muscles resulted in decreased range of motion of rearfoot eversion-inversion during running^15^. Hip abduction moments of force (i.e., in the hip frontal plane) are often emphasized to explain these results^5^; however, strengthening these muscles also increased hip adduction angles in that study^15^, which is contrary to the emphasis on hip abduction moments of force. In addition, they observed a trend toward a decrease in hip internal rotation^15^. The present results suggest that the reduction in pronation observed in the previous studies^15^ ^,^ ^33^ was influenced by changes in proximal transverse-plane mechanics.

We acknowledge that, in the present study, experimental measures of hip and knee mechanics could reinforce conclusions on the concept tested, since the bands were wrapped around both to the thigh and shank. However, it was not possible to measure hip passive moments since the appropriate methods available in the literature are executed with the knee joint flexed at 90º^34^, which would probably modify the band tension in comparison with walking. A measure of the passive knee transverse-plane moment, appropriate for this study, was also not available in the literature. Therefore, even though all participants reported a sensation that the band was pulling the limb into external rotation, we could not be certain of the additional external rotation moments. This limits the discussion about the kinematic changes as being a result of direct and passive mechanical effects or a result of changes in muscle activity due to sensory effects related to the use of the bands. Hip and knee kinematics were not measured either, since the bands significantly displaced thigh soft tissues and prevented using clustered tracking markers on this segment. Nevertheless, it should be noted that a previous case study indicated that a similar elastic band decreased hip internal rotation in weight-bearing tasks^16^, which was the effect expected at the hip in the present study and supports the observed relation between proximal transverse-plane and foot frontal-plane mechanics^2^.

The clinical extent of the results is very limited due to the methodological features of the study. The participants did not necessarily present excessive hip internal rotation and foot pronation, which are characteristics of the population that could benefit from the proposed interventions to increase external rotation moments. Thus, although the findings suggest the hypothesized kinematic relationship, it is not possible to draw any conclusions about the effects on people with excessive pronation and internal rotation. In addition, the proof of concept was conducted by means of an acute intervention and thus inferences about any mid- and long-term effects, during or after use of the bands, should not be made. Finally, no conclusions can be drawn on the effectiveness of the intervention for prevention or treatment of symptoms related to these kinematic patterns^5^ ^-^ ^8^.

The present findings suggest a possible contribution of proximal transverse-plane mechanics on rearfoot eversion-inversion during walking. Although the kinematic relationship indicated is still speculative, it agrees with previous investigations and theoretical propositions that explain rearfoot motion during walking^3^ ^,^ ^14^ ^,^ ^15^. The passive mechanical and/or sensory-active nature of the mechanisms that changed rearfoot motion and any possible clinical implications need to be subjected to further scrutiny by future studies.

## Conclusions

The use of external rotation elastic bands at the lower limb decreased rearfoot eversion during the stance phase of walking. The findings constitute preliminary experimental evidence on the relationship between the range of motion of rearfoot eversion, during walking, and proximal mechanisms that pull the lower limb into external rotation.
